# Influence of Sex and Age on Irisin Levels in Pediatric Type 1 Diabetes: A Pilot Study

**DOI:** 10.3390/jcm14030793

**Published:** 2025-01-25

**Authors:** Shay Averbuch, Oxana Gaiduk, Michal Yackobovitch-Gavan, Irina Laurian, Anna Dorfman, Gabi Shefer, Avivit Brener, Yael Lebenthal

**Affiliations:** 1 The Institute of Pediatric Endocrinology, Diabetes and Metabolism, “Dana-Dwek” Children’s Hospital, Tel Aviv Sourasky Medical Center, Tel Aviv 6423906, Israel; shayaverbuch@mail.tau.ac.il (S.A.); irinas@tlvmc.gov.il (I.L.); annado@tlvmc.gov.il (A.D.); avivitb@tlvmc.gov.il (A.B.); 2 Faculty of Medicine and Health Sciences, Tel Aviv University, Tel Aviv 6997801, Israel; oxangai@tlvmc.gov.il (O.G.); gabish@tlvmc.gov.il (G.S.); 3 The Endocrine Laboratory, The Institute of Endocrinology, Metabolism and Hypertension, Tel Aviv Sourasky Medical Center, Tel Aviv 6423906, Israel; 4 Department of Epidemiology and Preventive Medicine, School of Public Health, Faculty of Medicine, Tel Aviv University, Tel Aviv 6997801, Israel; michalyg2000@gmail.com; 5 Nursing Services, “Dana-Dwek” Children’s Hospital, Tel Aviv Sourasky Medical Center, Tel Aviv 6423906, Israel

**Keywords:** bioelectrical impedance analysis (BIA), body composition, glycemic control, irisin, partial clinical remission, pediatric patients, type 1 diabetes (T1D)

## Abstract

**Background**: Irisin is a myokine involved in the browning of adipocytes, the regulation of body composition and the enhancement of glycemic control. Additionally, irisin has been suggested to play a role in signaling mechanisms associated with the onset of puberty. In this study, we aimed to explore the interaction between muscle and adipose indices, urine irisin levels and glycemic control. **Methods**: This cross-sectional pilot study enrolled 76 consecutive pediatric patients (mean age 11.7 ± 3.8 years) diagnosed with type 1 diabetes (mean disease duration 2.1 ± 1.6 years). Body composition was assessed by bioelectrical impedance analysis (MFR z-score and skeletal muscle mass index). Urine irisin levels and glycemic control parameters (HbA1c, insulin dose-adjusted A1c [IDAA1c]) were evaluated. One linear regression model, stratified by sex, analyzed the sex-specific impact of puberty and age on irisin levels. A second linear regression model explored the associations of selected variables with irisin levels. **Results**: The first linear regression model revealed that irisin levels rise with age in prepubertal boys and decline with increased age among pubertal boys. The second linear regression analysis revealed no significant associations between irisin levels and metabolic parameters after adjusting for covariates. In contrast to boys, there were no significant interactions found in girls. **Conclusions**: Our novel findings revealed sex and age differences in the irisin levels of children and adolescents with type 1 diabetes. The dynamics underlying the role of irisin during pubertal development in the pediatric population with diabetes warrant further exploration.

## 1. Introduction

Irisin is a myokine that arises from the cleavage of fibronectin type III domain 5 (FNDC5) and is released by both adipose tissue and skeletal muscle following exercise [1]. Irisin is proposed to contribute to mediating some of the beneficial effects associated with physical activity, such as weight loss and thermoregulation [2]. Irisin also plays a role in the browning of adipocytes [3], the downregulation of adipogenesis and the reduction of lipid accumulation [4]. It has also been hypothesized to play a role in the hormonal signaling surrounding puberty in healthy children [5]. However, reports on this subject vary, with some studies concluding that irisin levels are higher in pubertal children [6], while others suggest that there is no variation in irisin levels across pubertal stages [7,8].

The influence of exercise on irisin levels can differ based upon factors such as the type, intensity and duration. Sporadic bouts of intense endurance or strength training have been associated with significant but transient increases in circulating irisin, typically peaking shortly after exercise [9,10,11]. Regular exercise may also influence FNDC5 and irisin levels, although responses can vary widely [11,12].

Some studies have linked irisin to the regulation of body composition and to cardiovascular and metabolic diseases in adults and in children [3,13]. However, other studies have yielded conflicting results regarding associations between irisin levels, blood pressure levels, fat mass and muscle mass in the pediatric population [3]. Some studies have reported elevated irisin levels in children with obesity compared to healthy children [14], suggesting an association between irisin levels, body mass index, weight class and waist-to-hip ratio [8,15].

Pediatric patients with type 1 diabetes exhibited higher irisin levels compared to healthy controls [16]. One study on the association between irisin and glycemic control in 96 pediatric patients with type 1 diabetes revealed a negative correlation between irisin levels and HbA1c, serum glucose levels and diabetes duration, suggesting a potential association with better glycemic control [16]. Following type 1 diabetes diagnosis and the initiation of insulin therapy, many patients experience partial clinical remission, characterized by improved glycemic control and lower exogenous insulin requirements due to a temporary recovery in beta cell function [17]. That phase has been linked to better long-term glycemic control [18,19,20] and to a reduction in lipid levels, which can lower the risk of cardiovascular complications [21,22]. Investigating the relationship between irisin and partial clinical remission may suggest its potential as a contributing factor during that phase.

Given the reported relationship between irisin levels and glycemic control [3], as well as the link between the muscle-to-fat ratio (MFR) and glycemic control [23,24,25], we hypothesized that there is a potential bi-directional relationship between irisin levels and MFR. Therefore, in addition to investigating the variables of sex and age in association with irisin levels, our pilot study also examined the interaction between muscle indices, urine irisin levels and glycemic control in pediatric patients with type 1 diabetes.

## 2. Materials and Methods

### 2.1. Study Design and Patients

Pediatric patients with type 1 diabetes who were being routinely followed-up on by our outpatient clinic were invited to participate. All children aged 5–18 years with a diabetes duration of <5 years, an available urine sample and same-day body composition assessment were eligible. The study protocol conformed to the Declaration of Helsinki principles (0950-20-TLV) and was approved by the institutional ethics review board. Written consent to participate was obtained from the parents of minors, while individuals aged ≥ 16 years signed an assent form. During 2022, 81 of the 82 consecutive eligible patients with type 1 diabetes agreed to participate in the study, and three adolescent girls were excluded due to menstruation at the time of their visit. The size of this pilot study was restricted due to the cost of FNDC5 ELISA kits. The results of 2 of the 78 urine samples that were assayed were not analyzed due to procedural errors. The 76 patients included in the study were stratified according to sex and pubertal stage.

### 2.2. Study Protocol

Each participant underwent a complete physical examination including anthropometric measurements, vital signs, pubertal stage according to Tanner, measurements of their capillary HbA1c levels (on a DCA 2000 device from Bayer Diagnostics Inc., Mississauga, ON, Canada, with a 95% confidence interval of 4.3–5.7%) and their body composition (bioelectrical impedance analysis [BIA]).

Data on physical activity were collected by means of self-reported questionnaires on daily activity levels. Physical activity was categorized as “adequate” if participants met the International Society for Pediatric and Adolescent Diabetes (ISPAD) recommendations of ≥60 min of daily physical activity, as shown in Table 1 and Table 2. While this measure provides a general indication of adherence to physical activity guidelines, it does not capture detailed information on the type, intensity or duration of exercise, which can influence irisin release. Due to these limitations and potential for self-reporting bias, physical activity was excluded from the linear regression models to minimize confounding and ensure a more robust analysis.

### 2.3. Body Composition Assessment

Since 2018, one of the therapeutic goals of our Diabetes Center has been to reduce modifiable cardiovascular disease risk factors. Patients with type 1 diabetes receive comprehensive glycemic control and metabolic risk assessment, including body composition measurement through bioimpedance analysis (BIA; Tanita Body-Composition Analyzer, Tanita MC-780 MA and GMON Professional Software, GMON Pro) during clinic visits as part of the routine standard of care [26]. The measurement takes a few minutes per subject and has been clinically verified as being accurate and reliable [27,28]. BIA measures both whole body and segmental (trunk, upper and lower limbs) fat and muscle. It also provides data adjusted for sex, age and height. Calculated BIA variables include appendicular skeletal muscle mass (ASMM; the sum of muscle mass of four limbs), MFR (the ratio between ASMM and fat mass) and a skeletal muscle mass index (the ratio between ASMM and height in meters squared). The sex- and age-specific reference curves for MFR in healthy white children and adolescents were used for calculation of the z-scores [29].

### 2.4. Urine Protein Analysis

Fresh urine samples were collected and stored at −80 °C until analysis. The samples were analyzed in duplicate by ELISA for fibronectin type III domain-containing protein 5 (FNDC5), a precursor of irisin by a chemistry analyzer (Cloud-Clone Corp., Katy, TX, USA), which has high sensitivity and specificity for the detection of FNDC5, with a 15.6–1000 pg/mL detection range and an intra-assay coefficient of variability of <10%.

### 2.5. Definition of Study Variables

Pubertal stage was determined according to the Marshall and Tanner criteria, which categorize physical changes during puberty into five stages (Tanner stages 1–5). Prepubertal individuals were classified as being in Tanner stage 1. Pubertal onset was defined as the appearance of Tanner stage 2 genitalia in boys, with a testicular volume of ≥4 mL, and the development of breast buds in girls [30,31]. The clinical remission phase was defined as insulin dose-adjusted A1C: IDAA1C [A1c (percent)] + [4 × insulin dose (units per kilograms per day)] ≤ 9. This definition, validated by a stimulated C-peptide level > 300 pmol/L, has shown a strong correlation with residual beta cell function and is therefore a useful tool in predicting partial remission [17]. Blood pressure percentiles were calculated with an online age-based pediatric BP calculator [32].

### 2.6. Statistical Analysis

All analyses were performed using Statistical Package for the Social Sciences software version 28 (SPSS Inc., Chicago, IL, USA). All performed statistical tests were two-sided. The Kolmogorov–Smirnov test or the Shapiro–Wilk test were performed to test the normality of continuous data. Data are expressed as means ± standard deviation (SD) for normally distributed variables and median and interquartile range [IQR] for skewed distributions. Comparisons between groups for continuous variables were made using independent sample *t*-tests for normally distributed data and Mann–Whitney *U* tests for skewed distributions. Categorical variables were evaluated by the chi-squared test or Fisher’s exact test as appropriate. One linear regression model, stratified by sex, was used to analyze the sex-specific impact of puberty and age on irisin levels. A second forward linear regression model was used to explore the associations of selected variables on irisin levels.

## 3. Results

The cohort consisted of 76 children and adolescents with type 1 diabetes, including 48 boys (63.2%) and 28 girls (36.8%). Of the total cohort, 36 participants (47.3%) were prepubertal. The mean age of the cohort was 11.7 ± 3.8 years, with a mean diabetes duration of 2.1 ± 1.6 years. At the time of assessment, the median HbA1c was 7.2% [IQR 6.7, 8.1]. A comparative analysis of metabolic parameters in pediatric patients with type 1 diabetes stratified by sex is provided in Table 1.

**Figure 1:**
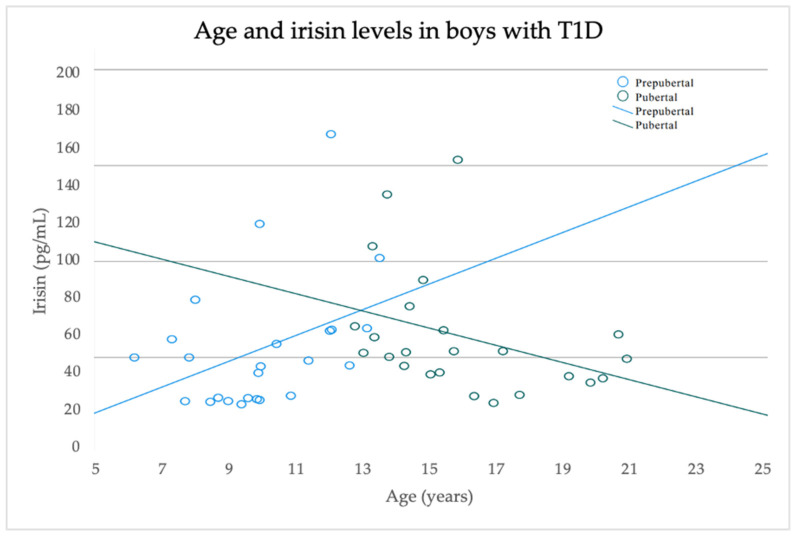
A graphical depiction of a linear regression model illustrating that irisin levels increase with age among prepubertal boys and decrease with age among pubertal boys.

The pubertal boys with type 1 diabetes were characterized by significantly higher skeletal muscle mass indices (*p* < 0.001), MFR z-scores (*p* = 0.004) and creatinine levels (*p* = 0.001) compared to the prepubertal boys (Table 2). A linear regression model, stratified by sex, was used to analyze the sex-specific impact of puberty and age on irisin levels. That model found a significant interaction between age and puberty (β= −11.0, SE = 4.2, *p* = 0.011), while irisin levels rose with increasing age among prepubertal boys, whereas irisin levels declined as age increased among pubertal boys (Table 3, Figure 1). Following these results, a second forward stepwise linear regression analysis was conducted to investigate the relationship between irisin levels and metabolic parameters. First, sex and age were entered into the model as covariates to control for their potential confounding effects. In the second step, the added parameters included the duration of diabetes and the HbA1c, IDAA1c, creatinine, skeletal muscle mass index, MFR z-score and blood pressure levels. The model found no significant associations.

Pubertal girls with type 1 diabetes were characterized by significantly higher skeletal muscle mass indices (*p* < 0.001) and creatinine levels (*p* = 0.007) than prepubertal girls (Table 2). Unlike boys, no significant interaction was found between puberty and age in girls. Additionally, no significant association with irisin was found in the model which included duration of diabetes, HbA1c, IDAA1c, creatinine, skeletal muscle mass index, MFR z-score and blood pressure levels.

## 4. Discussion

To the best of our knowledge, this pilot study is the first to examine interactions between irisin levels in different pubertal stages among pediatric patients with type 1 diabetes. Our findings revealed sex differences in the interaction between irisin and age, with age-related trends differing by pubertal stage solely among boys. Specifically, irisin levels increased as age advanced in prepubertal boys and decreased as age advanced in pubertal boys. There were no comparable pubertal stage-related differences in irisin levels among girls with type 1 diabetes.

Irisin has been hypothesized to play a role in the signaling mechanisms associated with the activation of the hypothalamic–pituitary–gonadal axis and the onset of puberty [5]. Our evidence of the rise in irisin with increasing age among prepubertal boys together with the opposite trend in pubertal boys supports this hypothesis. Interestingly, studies in rodents and primates have shown this mechanism to be more dominant in girls [33,34,35]. The difference between sexes may suggest divergent biological mechanisms with specific roles of estrogen and testosterone, although the limited sample of girls may have also contributed to this difference in our study.

Irisin has been associated with a reduction in insulin resistance [36]. An alternative explanation for the elevated irisin levels within the male prepubertal subgroup could be related to the physiological insulin resistance observed preceding and during puberty [37,38]. The onset of puberty in individuals with obesity is characterized by a supraphysiological rise in insulin resistance, which was associated with an increase in irisin levels [6], highlighting the relationship between insulin resistance and irisin levels in humans. This rise in irisin levels, which was potentially triggered by heightened insulin resistance, may contribute to the patterns observed in our study.

There is contradictory evidence regarding the association between irisin levels and cardiometabolic parameters in the pediatric population. Previous studies have indicated that irisin levels are correlated with cardiometabolic parameters such as blood pressure and BMI, suggesting the potential use of irisin as a surrogate marker for metabolic syndrome [13,39,40]. Conversely, other studies did not find significant associations between these factors [3,41]. Our study aligns with the latter group, providing specific insights into the pediatric population with type 1 diabetes, as we found no association between irisin levels and body composition parameters or blood pressure levels.

We analyzed boys and girls separately in accordance with the findings of studies that documented substantial sex differences in body composition among individuals with type 1 diabetes. Girls with type 1 diabetes were reported to have higher fat and truncal fat percentages, along with lower MFR z-scores compared to boys [23,24]. Furthermore, sexual dimorphism was observed in glycemic control, with girls exhibiting higher HbA1c levels at diagnosis as well as over time [42]. They also reportedly spend less time within the optimal blood glucose range [43]. These findings underscore the importance of sex-specific approaches in managing pediatric patients with type 1 diabetes to optimize clinical outcomes.

Although they are preliminary, our findings suggest potential pathways for improving the clinical understanding of metabolic parameters in children and adolescents with type 1 diabetes. Further investigations into the relationship between irisin levels, glycemic control and metabolic markers may help in guiding the development of more personalized treatment strategies. They could also provide insights into insulin requirements and the early markers of cardiovascular risk, ultimately contributing to the better management of type 1 diabetes in pediatric patients.

Our study has certain limitations. The main ones are related to the small sample size, which necessitates caution when interpreting our findings, and the cross-sectional design, which prevents the establishment of causal relationships. Another limitation is that it may not be generalizable to the entire pediatric population with type 1 diabetes, since the sample comprises children attending a single pediatric diabetes center. Furthermore, physical activity data were excluded from the analysis due to their being self-reported, despite the importance of examining associations between physical activity and irisin. A key strength of this study is the uniformity of medical care delivered by a multidisciplinary team within a single hospital-based diabetes center, as well as the novel approach of exploring the interaction between muscle indices, urinary irisin levels, glycemic control and puberty in this population.

In conclusion, our preliminary findings highlight sex and age differences in irisin levels among children and adolescents with type 1 diabetes. The absence of interactions between urinary irisin levels, muscle indices or glycemic control call for further research to clarify the potential role of irisin in metabolic and hormonal processes during different stages of pubertal development in children and adolescents with diabetes.

## Figures and Tables

**Table 1:** Comparative analysis of metabolic parameters in pediatric patients with type 1 diabetes stratified by sex.

	Boys, *n* = 48	Girls, *n* = 28	*p* Value
Age, years	12.1 ± 3.8	11.1 ± 3.6	0.283
Systolic blood pressure (%)	61.0 [41.8, 82.0]	73.2 [53.0, 83.8]	0.365
Diastolic blood pressure (%)	58.6 [46.2, 70.7]	67.6 [56.4, 79.5]	0.118
Adequate physical activity, *n* (%)	25 (52.1)	17 (60.7)	0.470
Fat, %	17.3 [15.4, 24.0]	25.5 [22.7, 28.5]	**<0.001**
Truncal fat, %	14.2 [11.2, 19.3]	18.7 [16.6, 23.5]	**0.001**
ASMM	14.9 [8.5, 19.4]	11.9 [7.4, 14.6]	**0.027**
ASMM z-score	0.03 [−0.53, 0.50]	−0.25 [−0.76, 0.66]	0.474
Skeletal muscle mass index	5.8 [4.6, 6.6]	4.8 [4.4, 5.6]	**0.038**
MFR	1.62 [1.19, 2.28]	1.10 [0.90, 1.23]	**<0.001**
MFR z-score	−0.30 [−1.24, 0.24]	−0.59 [−0.98, −0.28]	0.296
HbA1c, %	7.4 [6.7, 8.2]	7.1 [6.8, 7.8]	**0.850**
IDAA1c	10.3 [9.3, 11.7]	10.6 [9.2, 11.7]	0.817
Urine irisin, pg/mL	50.0 [35.3, 64.2]	61.3 [37.7, 88.9]	0.275
Serum creatinine, mg/dL	0.50 [0.43, 0.64]	0.45 [0.40, 0.61]	0.274

Data are presented as number (percent), mean ± standard deviation or median [interquartile range]. The Mann–Whitney U test was performed to test non-parametric medians. Self-reported physical activity was considered adequate if it met the ISPAD recommendations of ≥60 min each day. *n*, number; ASMM, appendicular skeletal muscle mass; MFR, muscle-to-fat ratio; HbA1c, hemoglobin A1c; IDAA1c, insulin dose-adjusted HbA1c. Bold indicates significance.

**Table 2:** Comparative analysis of metabolic parameters in pediatric patients with type 1 diabetes stratified by pubertal status.

	Boys, *n* = 48			Girls, *n* = 28		
	Prepubertal, *n* = 24	Pubertal, *n* = 24	*p* Value	Prepubertal, *n* = 12	Pubertal, *n* = 16	*p* Value
Age, years	9.0 ± 2.0	15.2 ± 2.6	**<0.001**	7.5 ± 1.4	13.9 ± 1.9	**<0.001**
Systolic blood pressure (%)	64.0 [50.0, 86.1]	56.5 [25.4, 80.5]	0.183	73.9 [56.5, 84.7]	70.3 [48.8, 81.9]	0.329
Diastolic blood pressure (%)	62.2 [44.6, 77.8]	56.8 [46.6, 66.0]	0.695	70.8 [57.3, 79.5]	66.0 [53.6, 79.5]	**0.010**
Adequate physical activity, *n* (%)	9 (37.5)	16 (66.7)	**0.045**	5 (41.7)	12 (75)	0.079
Fat, %	20.0 [17.2, 25.4]	15.9 [12.4, 17.8]	**0.002**	25.3 ± 3.9	26.6 ± 5.6	0.516
Truncal fat, %	14.9 [12.6, 20.5]	11.7 [9.0, 17.0]	0.076	19.6 ± 4.4	20.5 ± 6.1	0.692
ASMM	8.5 [6.8, 10.1]	19.6 [16.9, 25.3]	**<0.001**	7.2 ± 1.9	14.2 ± 2.0	**<0.001**
ASMM z-score	0.21 ± 0.85	0.31 ± 0.74	**0.029**	−0.18 ± 0.78	−0.11 ± 1.03	0.842
Skeletal muscle mass index	4.7 ± 1.2	6.8 ± 1.1	**<0.001**	4.4 ± 0.4	5.5 ± 0.7	**<0.001**
MFR	1.3 [1.1, 1.6]	2.3 [2.0, 2.9]	**<0.001**	1.1 ± 0.2	1.1 ± 0.3	0.629
MFR z-score	−0.8 ± 0.7	0.2 ± 1.4	**0.004**	−0.82 ± 0.50	−0.36 ± 0.76	0.076
HbA1c, %	7.1 [6.7, 8.0]	7.6 [6.3, 8.2]	0.718	7.0 [6.8, 8.4]	7.2 [6.8, 7.6]	0.944
IDAA1c	9.8 [9.3, 11.2]	10.8 [8.9, 11.9]	0.398	10.3 [9.1, 11.8]	10.6 [9.2, 11.6]	0.944
Urine irisin, pg/mL	47.1 [28.7, 64.0]	52.3 [40.9, 64.7]	0.255	61.2 [35.1, 70.8]	86.9 [49.0, 113.3]	0.180
Serum creatinine, mg/dL	0.47 [0.39, 0.50]	0.65 [0.47, 0.75]	**0.001**	0.41 ± 0.12	0.44 ± 0.12	**0.007**

Data are presented as number (percent), mean ± standard deviation or median [interquartile range]. The Mann–Whitney U test was performed to test non-parametric medians. Self-reported physical activity was considered adequate if it met the ISPAD recommendations of ≥60 min each day. *n*, number; ASMM, appendicular skeletal muscle mass; MFR, muscle-to-fat ratio; HbA1c, hemoglobin A1c; IDAA1c, insulin dose-adjusted HbA1c. Bold indicates significance.

**Table 3:** A linear regression model, in boys, analyzing the impact of puberty and age on irisin levels.

	β	Std. Error	t	Sig.	95% Confidence Interval		Partial Eta Squared
					Lower Bound	Upper Bound	
Intercept	127.5	38.8	3.29	0.002	49.4	205.7	0.197
Puberty	132.5	49.4	2.68	0.010	33.0	232.0	0.141
Age	−4.4	2.5	−1.75	0.088	−9.49	0.681	0.065
Puberty × Age	−11.0	4.2	−2.6	0.011	−19.4	−2.61	0.137

Parameters entered into the model were puberty, age and irisin levels.

## Data Availability

The data used to support the findings of this study are available from the corresponding author upon reasonable request.
